# Atlanta Society of Medicine

**Published:** 1894-06

**Authors:** R. R. Kime

**Affiliations:** Secretary; 63½ Whitehall St.; Atlanta, Ga.


### *SOCIETY REPORTS.*

### ATLANTA SOCIETY OF MEDICINE.

ATLANTA, GA., APRIL 17, 1894.

*President J. B. Duncan, M.D., in the Chair.*

### CHOLERA INFANTUM.

Discussion opened by R. R. KIME, M.D.

*Causes.*—This is a very interesting subject, especially as we are soon to reach the period at which cholera infantum makes its annual visits to claim its victims from many households and sadden many hearts. As its visitations are annual, and at certain seasons, there must be conditions and forces peculiar to such seasons that cause or favor its development. Attacks being unknown in the winter, but common in the hot season, it is evident that elevated temperature is either a direct or predisposing cause.

Some authorities claim it to be the direct cause, and that cholera infantum is but sun-stroke, coup de soleil or heat stroke. This view is scarcely tenable, as we will see by a comparison of the two diseases:

| CHOLERA INFANTUM. | SUN-STROKE. |
|---|---|
| Temperature range high, 105° to 108° F. | The same. |
| Great heat of head, contracted pupils, thin fecal evacuations, embarrassed respirations and cerebral symptoms common toward the close. | Common to sun-stroke, but appear more rapidly. |
| Gastro-intestinal symptoms more prominent and precede. | Less prominent and later. |
| Attack comes on more gradual. | Attack *sudden.* |
| Errors in diet and diarrhea favor attacks. | No direct connection. |
| At onset infant often wide-awake, restless, thirsty. | Drowsiness and stupor common. |
| May occur at any hour or any day during the hot season. | Only during hottest part of the day. |
| May occur where sun-stroke is scarcely known. | *Cholera infantum always* occurs where sun-stroke occurs. |
| Gastro-intestinal catarrh present and the essential lesion. | Rarely present. |
| Often due to absorption of ptomaïnes, including tyrotoxicon, etc. | Never a direct cause. |

Neither can we conceive of cholera infantum being due to brain lesions; while there are some symptoms that indicate involvement of the brain, they are but secondary, being the result, and not the cause, of the disease.

If we would prevent cholera infantum, we must look well to the hygienic surroundings and dietary of infants, removing from high temperatures, when possible, such children as have conditions predisposing to the disease.

While cholera infantum is not due to a specific micro-organism, yet many cases are due to the absorption of an alkaloid—ptomaïnes—due to the presence of germs, which are often saprophytic in character.

This being true, the toxicogenic germs are capable of producing the disease as well as the specific pathogenic germs; hence we should endeavor to prevent, as far as possible, their entrance into the alimentary canal. This is one reason why the mother's milk should be given preference in infant dietary.

There are so many sources by which the food may be contaminated, where children depend upon artificial food for nourishment, that it plays no minor part in the causation of cholera infantum. The adulteration, fermentation and improper keeping of milk unfit it for use by the infant. Also the manner in which it is given, not sterilized, out of vessels containing germs of putrefaction, with infected bottles and rubber tubes carrying death in proportion to its length.

When nature's life-giving lacteal fluid is imbibed from a mature, healthy mother, it is as near aseptic as it is possible to obtain it, and of itself not likely to cause disease. To render artificial foods harmless, they not only should contain the necessary tissue-forming substances, but should be aseptic, if not rendered so by sterilization, and kept so by proper containers, and given to the infant in sterilized bottle, with a rubber nipple properly sterilized.

While improper food, introduction of germs, increased heat and unsanitary surroundings will not individually of themselves always produce cholera infantum, in a weakened, debilitated infant, with gastro-intestinal irritation and indigestion, or even a previously healthy infant, with acute intestinal catarrh, these influences may at any time precipitate an attack.

We conclude that the primal cause of cholera infantum in most cases is a catarrhal condition of stomach and intestinal canal, coupled with increased heat and improper food, or absorption of ptomaïnes, to which in many instances may be added unsanitary surroundings.

*Pathology.*—(By E. H. Richardson, M.D.)—*Mr. President:* In obeying your request to open the discussion on the pathology of cholera infantum, at the threshold of my investigations, I am confronted with the dictum of no less an authority than DaCosta, that "the exact pathology of the disease is unknown." This declaration from so distinguished a source is far from encouraging; and in the endeavor to ascertain the truth on the subject, additional obstacles obstruct our research and admonish us to refrain from infringing upon the etiology of the disease and to avoid invading the domain of conjecture and speculation. I think the clinical history of the malady warrants the belief that in teething children the intense heat of summer, coupled with an impure atmosphere and improper feeding, causes to be generated within the body an irritant poison—possibly some of the ptomaïnes—which has an elective affinity for the primæ viæ and the sympathetic nerve. In the present state of our knowledge of course it is impossible to say definitely whether this poison is produced within or outside the alimentary canal. But that it acts as a violent sedative to the nerve centers, especially disturbing the great sympathetic system of nerves and the viscera over which it presides, we feel absolutely sure. The advent of this poison into the alimentary canal of the child means havoc to the subject, very rapidly producing prostration and collapse and transudation of the watery portion of the blood into the intestines and the stomach; and when death is not produced within a few hours, initiating an acute inflammation of the mucous membrane of the intestines, involving in some cases the mucous membrane of the stomach also, producing a very high blood temperature (rectal temperature running from 105 to 107). In observing the phenomena of this disease we note the *apparent* absence, in its gravest type, of one of the characteristic symptoms of inflammatory diseases, to wit: pain. When death occurs within a few hours from this terrific malady, like the trackless arrow that passes through the skies, it leaves no trace behind. "It is well known to pathologists that in inflammations of the mucous surfaces of short duration the redness is apt to disappear in the cadaver." All pathologists concede that inflammation and its sequelæ of the mucous membrane of the intestines is the essential primary local lesion of *cholera infantum*. The gastro-intestinal lesions observed in the subjects of those dying in the first stages of the disease are congestion and hyperemia of the mucous membrane of the ileum and colon, with hyperplasia of the solitary and mesenteric glands. The whole intestinal tract is occasionally involved, softening of the follicles almost constantly found. Where the disease has existed for some time and resulted in an entero-colitis, follicular ulcers are present in the small intestine, and sometimes in the large intestine. The liver sometimes is found congested and engorged. When the disease has been chronic inflammation of the meninges of the brain, especially of the arachnoid membrane with congestion of the cranial sinuses and effusion into the ventricles is found to have existed, or a secondary manifestation of the disease.

*Symptoms and Treatment.*—(By C. D. Hurt, M.D.)—The symptoms of cholera infantum may be denominated usual or common, and unusual or rare. They may also be denominated malignant or benign. They are also dependent upon the age of patient and stage of the disease. Usually cholera infantum is ushered in by the violent vomiting, which may or may not be attended in the outset with diarrhea, but the diarrhea quickly follows. The action of the patient in the effort of vomiting, or I should say the action of the stomach, is usually characteristic of the presence of this dreadful malady; that is to say, when emesis comes on it is the result of a very violent spasm of the stomach, which ejects its contents in a manner suggestive of the forcible action of a syringe. Sometimes cholera infantum has what may be termed a prodromic stage. The patient is a little dull, loss of appetite, more or less pallor, possibly a little disturbance of the bowels in the form of diarrhea; this condition may last indefinitely, but if followed by cholera infantum it usually comes on in from twenty-four to seventy-two hours. Sometimes the first symptom is vomiting, then again the first symptom is constant colliquative discharges from the bowels. Usually fever of the intermittent type is present, the surface is cold, while a thermometer in the mouth or in the rectum measures a temperature of 102 or 104½. Coldness of the surface is therefore not indicative of the true temperature. The vomiting becomes very persistent and frequent, and the stomach rejects everything in the way of food, or even cold water. With this continued waste, both by vomiting and purging, followed by a shock upon the nervous system, as well as the whole constitution, it is very readily recognized after it has existed for a few hours. Later on the tissues of the body waste very rapidly, the child becomes very weak and the skin very flabby, its eyes sunken and countenance very pale. Attending this vomiting and purging there are usually very severe abdominal pains, causing the child to cry out. Sometimes there are cerebral complications, which are liable to develop constantly as the action of the kidneys may be interfered with. When we are called to a case of cholera infantum that has existed for several days we find general exhaustion, attended with emaciation and anemia, all tending to rapid dissolution. The disease usually lasts but a few days before fatal results follow, unless the disease is checked and recovery begins. There are, however, some cases which seem to assume chronic form, and which become complicated with entero-colitis. While in this condition the stomach has grown more tolerant of food and does not reject all, so that some nourishment is retained and assimilated.

Now as to treatment. When called to a child in the first stage of cholera infantum, whether vomiting and purging, one or both are manifest, we should endeavor by the most speedy and safe method to eliminate the causes and arrest the effect, assuming the causes to be such as have been already mentioned by Dr. Kime, who preceded me in his remarks—that is to say, the characteristic bacilli of cholera infantum. We endeavor in our treatment to destroy all these germs and to prevent any new development. While our efforts are extended in this direction, it is also well to take some notice of the effects which are already manifest upon the system and meet them with proper treatment. Quite a number of remedies are found in our vocabulary and many advocates for each. That which I have found most satisfactory is the administration of a small quantity of mercury, preferably in the form of calomel, say one-tenth to one-twelfth of a grain, combined with a little pulverized opium, and from one to three grains of bicarbonate of soda. A powder of such dimensions, given from one to four hours, according to the urgency of the case, will likely arrest vomiting, and so change the character of the stools as to show the presence of bile, and less of a liquid nature. This course of treatment may be pursued from twelve to twenty-four hours before the results are altogether satisfactory. Added to this we might use a cold water application, or the reverse is sometimes beneficial, a warm poultice over the abdomen and stomach. We should also endeavor to warm up the extremities. This is accomplished by the administration of warm mustard baths, repeated from two to four hours. The ordinary chalk and opium powders, given in doses of four grains, combined with one-tenth or one-twelfth of a grain of calomel, proves very effective, in first, arresting vomiting; second, in quieting the nervous system and inducing sleep; third, in checking the bowels; fourth, in so altering the secretions as to bring about healthy stools. I have had much experience with this latter prescription, with very happy results. I sometimes use the bichloride of mercury, combined with a colorless solution of hydrastis. 1-100 of a grain of the former and five drops of the latter, repeated from one to three hours, according to the urgency. The bichloride has the property of lessening pain, checking the bowels as well as vomiting, and largely supersedes, if not entirely obviates, the need of opium. I have found this, however, better adapted to milder cases, where the vomiting recurs from two to four hours, with the ordinary severe diarrhea of the bowels. I have also had some experience with the arsenite of copper, repeated at short intervals, and in very minute doses, say 1-5000 of a grain, given in solution hourly. Nitrate of silver is recommended. I have had but little experience, and cannot speak as to its value. Stimulants are demanded in nearly all cases. It is a mistake to delay their use too long. Brandy is a preferable form, given in ten to twenty-drop doses properly diluted. Bismuth, creosote and the vegetable astringents have all been used largely. The first of these, I am satisfied, is a good absorbent, somewhat antispasmodic and astringent; combined with mercury and opium it serves a valuable purpose. The vegetable astringents I cannot indorse. I have failed to receive any benefit, and have been disappointed and lost valuable time waiting upon their use. I would recommend more heroic treatment. After the violence of the disease has been checked, and the destruction of the bacilli or germs of cholera, we might then depend more or less upon these milder remedies. I have not mentioned the removal of the child from the unhealthy surroundings. This of course should in every instance be attended to. If it is in a marshy, malarial district, it should be removed to a drier, healthier atmosphere. If it is in a poorly ventilated dwelling, it should be removed to more healthy quarters. Change is beneficial, even from one healthy district to another. Another very essential thing is to guard well the changes in the temperature of the atmosphere. The circulation being weak, the extremities more or less cold anyway, should be protected against dampness and chilly mornings by the proper use of flannels. In other words, the peripheral circulation should be heartily encouraged by all means possible and safe.

Now as to the last, though not the least, in importance in all treatment, we come to that much disputed question, diet. Nature's nourishment in the form of mother's milk, coming from a healthy woman, cannot in any instance be improved upon, but unfortunately there are many children who are early forced from the breast, or who, on account of reasons uncontrollable, have to be raised from the bottle. The child thus dependent upon artificial means, has its way of successful growth and development to adult life largely hedged in. Its first summers, together with the work of dentition, have strewn the pathway with many victims. It is no easy matter for us to exercise hope, patience and perseverance when the little fellow is constantly rejecting all nourishment put into its stomach. We try the cow's milk, the condensed milk and the dozens of baby foods, with and without the presence of a pancreatin. We resort to the teas and the beef juices, and the whole catalogue of artificial nourishment, but, alas, have we not too often hurried by impatience into a too rapid succession of these varied forms of nourishment. I believe that the cow's milk, properly diluted, and given in proper quantities at set intervals, may be most relied upon; and just at this place I desire to say that I believe that much of our trouble in overcoming this disease is due to overfeeding, either in quantity or quality, or both. So much do I believe this, that I make it a rule to order the nourishment given at stated intervals in measured quantities, and to begin with it well diluted, depending somewhat upon the age of the child and the weakened condition of the stomach.

I think that the best brands of condensed milk are not to be condemned and discarded when properly diluted, and I believe that the only way of satisfactorily diluting them is by weight rather than measurement; it being a very heavy liquid or semi-solid, can be heaped upon a spoon in such a manner as to make the quantity two or three times what is needed or ordered. Even the most intelligent mothers and nurses often go amiss on this line. When cholera infantum is complicated with entero-colitis or dysentery, I think enemas of starch with a little laudanum, occasionally repeated, are very beneficial. Some advocate the liberal flushing of the colon with tepid water. This I think does good in some instances.

GENERAL DISCUSSION.

DR. PARKS: The subject has been well presented to-night. Dr. Holt claims three per cent. of bowel troubles as cholera infantum. Think we have but few cases of genuine cholera infantum, and they usually die. I often give hot water freely, repeated frequently, even if vomited. Prefers peptonized milk—making juncket, etc.

DR. STEPHENS: My treatment has been unsatisfactory; have tried various methods; flushes colon with hot water, adding salt; give hot water to drink, lime water; mercuric chloride; use stimulants early and freely. Believes small doses of medicine act best; uses poultices in convulsions. Extreme cases of collapse, use hot applications to head.

DR. COLLINS: Did not wish to say anything, but wished to emphasize the necessity of care in diet and feeding, as presented by Dr. Hurt.

DR. GRANDY: The term *cholera infantum* is objectionable, *entero-colitis* or *ileo-colitis* being preferable. Main cause is improper food, whether improper in the first instance or improper by fermentative changes after being introduced into alimentary canal. Best indication for treatment is to furnish proper food as near as possible. In absence of mother's milk, best substitute is cow's milk, prepared according to the age of the child. Some medicinal treatment will often be called for, and here is the indication for intestinal antiseptics. Have used in several cases the following combination: Bismuth subnitrate and salol, of each gr. ij, calomel, gr. $\frac{1}{8}$; one such powder every three hours to child two years old. In a late case had used with good result:

| | | |
|---|---|---|
| ℞ | Salol | gr. xlviii. |
| | Tr. op. camph | ʒ ij. |
| | Syr. rhei. aromat | ℥ i. |
| | Essence pepsin (Fairchild's) | qs. ad. ℥ iij. |
| M. | Sig.: Teaspoonful every three hours. | |

DR. GLASS, in treatment, condemns the use of opiates. They do more harm than good. Opiates confine the offending material in the alimentary canal, which is deleterious. Use mercuric chloride; it acts well and relieves pain. Advises stimulants. Uses brandy, but prefers sour wine.

DR. ARCH AVARY: We may have mild and severe. Seen but few cases as described in books, and they die promptly. One of the most prersistent symptoms is thirst. Stimulation offers hope—antiseptics essential. Thinks hypodermic medication offers much. Strychnia, morphia and then mercuric chloride, stimulants and food.

DR. F. B. MCRAE: All agree on causation. I give stimulants alone and sedatives alone. Give calomel first, not with an opiate; later stimulants, and, if necessary, opiates. Thinks mercury one of the best. Stimulate salivary secretions; uses carbolic acid in emulsions. Relies on antiseptic treatment, good nourishment. Uses turpentine to abdomen and thorax. Do not agree as to fatality expressed to-night. Gives carbolic acid in one to ten drops in emulsion. Gives brandy as stimulant.

DR. AMSTER: Would add that ignorance of mothers in feeding children, nursing too frequently and too freely, is a great cause of these troubles. Thinks ice water irrigations, intestinal, promises good result, carrying off poisons, etc. Would not discard opiates.

DR. STOCKARD: Uses free intestinal irrigations, hot or cold, as indicated. Sulpho-carbolate of zinc, after calomel and irrigations, is one of the best antiseptics.

DR. DUNCAN: Believes that the central lesion is in gastro-intestinal canal, nerve-centers paralyzed, causing free water discharges. Have grades of severity, from light to severe attacks, and treatment must vary as to the stage of the disease. Gives calomel, lactopeptine and soda. Allows them all the hot water they can drink. Flushes colon with warm salt water. In milder cases, gives mercuric chloride with pepsin. Does not use bismuth in cholera infantum. If used, prefers salicylate. Uses opiates in small doses. Diet essential—mother's milk preferable. Uses prepared foods. Malted milk good where child will take it. Thinks liver often at fault and advises calomel and mercuric chloride.

DR. RICHARDSON thinks wet nurse and change of air the best treatment. Believes in irrigation of colon. Thinks the hypodermic treatment an ideal method, but had not tried it.

DR. HURT: As to causes, they are various; extreme heat, change from mother's breast to artificial food, ingestion of improper food, foreign bodies. Believes teething a cause. By reflex action frees gums; cuts gum often freely, and as to opiates may have an anemic condition of brain. Where renal secretion is checked opiates are dangerous. Believes in irrigation, hot water by mouth. Been much disappointed in pepsins, peptonizing milk, etc. Uses stimulants.

R. R. KIME, M.D., Secretary.

63½ *Whitehall St.*

---

### ARSENIC IN PERNICIOUS ANEMIA.

Dr. E. A. Barton (*Lancet*) reports the following case of pernicious anemia which was relieved by the prolonged administration of arsenic. The patient, a coachman, exhibited all the usual signs of pernicious anemia, and a careful examination of the blood showed that the corpuscles numbered only eighteen per cent. The corpuscles varied in size, were soft and plastic, and formed rouleaux readily. There was no leucocytosis. He complained of being unable to read, and examination showed large hemorrhages into the retinæ. Five minims of Fowler's solution of arsenic were given thrice daily, together with a generous diet. The week after the arsenic treatment was begun the patient had a fainting fit, and the next morning he volunteered the statement that he saw red color when looking at anything white. A fortnight after the commencement of the arsenic there was no further bleeding from the gums; the patient declared himself stronger and was able to move about the room. In three weeks he was able to walk half a mile without fatigue, and in a month returned to work. The corpuscles on January 16, being seventy-six per cent. and the hemoglobin sixty per cent. The hemorrhages into the retinæ had entirely disappeared, except for a slight blur on the right side. He continued the use of the arsenic for ten weeks from the commencement.—*N. Y. Medical Times.*

